# Case-based reported mortality associated with laboratory-confirmed influenza A(H1N1) 2009 virus infection in the Netherlands: the 2009-2010 pandemic season versus the 2010-2011 influenza season

**DOI:** 10.1186/1471-2458-11-758

**Published:** 2011-10-04

**Authors:** Rianne AB van Gageldonk-Lafeber, Rob M Riesmeijer, Ingrid HM Friesema, Adam Meijer, Leslie D Isken, Aura Timen, Marianne AB van der Sande

**Affiliations:** 1 National Institute for Public Health and the Environment (RIVM), Centre for Infectious Disease Control (CIb), Bilthoven, the Netherlands; 2 Utrecht University Medical Center, Julius Centre, Utrecht, the Netherlands

## Abstract

**Background:**

In contrast to seasonal influenza epidemics, where the majority of deaths occur amongst elderly, a considerable part of the 2009 pandemic influenza related deaths concerned relatively young people. In the Netherlands, all deaths associated with laboratory-confirmed influenza A(H1N1) 2009 virus infection had to be notified, both during the 2009-2010 pandemic season and the 2010-2011 influenza season. To assess whether and to what extent pandemic mortality patterns were reverting back to seasonal patterns, a retrospective analyses of all notified fatal cases associated with laboratory-confirmed influenza A(H1N1) 2009 virus infection was performed.

**Methods:**

The notification database, including detailed information about the clinical characteristics of all notified deaths, was used to perform a comprehensive analysis of all deceased patients with a laboratory-confirmed influenza A(H1N1) 2009 virus infection. Characteristics of the fatalities with respect to age and underlying medical conditions were analysed, comparing the 2009-2010 pandemic and the 2010-2011 influenza season.

**Results:**

A total of 65 fatalities with a laboratory-confirmed influenza A(H1N1) 2009 virus infection were notified in 2009-2010 and 38 in 2010-2011. During the pandemic season, the population mortality rates peaked in persons aged 0-15 and 55-64 years. In the 2010-2011 influenza season, peaks in mortality were seen in persons aged 0-15 and 75-84 years. During the 2010-2011 influenza season, the height of first peak was lower compared to that during the pandemic season. Underlying immunological disorders were more common in the pandemic season compared to the 2010-2011 season (p = 0.02), and cardiovascular disorders were more common in the 2010-2011 season (p = 0.005).

**Conclusions:**

The mortality pattern in the 2010-2011 influenza season still resembled the 2009-2010 pandemic season with a peak in relatively young age groups, but concurrently a clear shift toward seasonal patterns was seen, with a peak in mortality in the elderly, i.e. ≥ 75 years of age.

## Background

In 2009, the rapid spread of an emerging influenza virus, A(H1N1) of swine origin, resulted in the first pandemic of the 21^st ^century [1]. This pandemic influenza A(H1N1) 2009 virus has led to a limited outbreak in the Netherlands with, as in many other countries, generally mild illnesses in the majority of patients [2,3]. The pandemic was considerably less lethal than was expected, with a low overall case fatality rate [4,5]. Nevertheless, a considerable part of the pandemic influenza related deaths concerned relatively young persons (mainly young and middle aged adults) [3,5-8]. This is contrary to seasonal influenza epidemics, where deaths occur mainly amongst elderly aged 65 years or older [9-11].

In the Netherlands, all deaths associated with laboratory-confirmed influenza A(H1N1) 2009 virus infection had to be notified since 30 April 2009. This mandatory notification remained in place during the influenza season 2010-2011, which in Europe has been characterized predominantly by the influenza A(H1N1) 2009 virus, and to a lesser extent influenza virus type B [12,13].

The national notification system provided detailed information of the clinical characteristics of all deaths associated with a laboratory-confirmed influenza A(H1N1) 2009 virus infection in the Netherlands. We performed a retrospective analysis of all fatalities, comparing the 2009-2010 pandemic season with the 2010-2011 influenza season, aiming to assess whether and to what extent pandemic mortality patterns concerning age distribution and underlying conditions were reverting to seasonal patterns.

## Methods

### Data collection

In the Netherlands, laboratory investigation was indicated for all hospitalised and/or deceased patients with suspected influenza A(H1N1) 2009 virus during the 2009-2010 pandemic as well as the 2010-2011 influenza season. Following laboratory confirmation of influenza A(H1N1) 2009 virus infection, name and clinical characteristics of hospitalised and deceased patients had to be reported to the municipal health service (MHS) by both the attending medical doctor and the head of the involved microbiology laboratory. The MHS entered the notifications into a national anonymous and password-protected web-based database, including structured questions about patient demographics and information on underlying medical conditions, treatments, clinical presentation, and admission to an intensive care unit (ICU). In the pandemic season 2009-2010, additional information on underlying conditions for deceased patients was collected by the Centre for Infectious Disease Control (CIb) of the National Institute for Public Health and the Environment (RIVM) in consultation with the MHS and subsequently added to the notification database.

### Data analysis

The notification database was used to perform a comprehensive analysis of all deceased patients with a laboratory-confirmed influenza A(H1N1) 2009 virus infection. Ethical approval was not required for this study as only anonymous data were used, and no (medical) interventions were made on human subjects.

Based on available clinical data, the underlying medical conditions were classified into nine groups: no underlying disorders, respiratory disorders, immunological disorders (including haematological malignancies), neurological disorders, intellectual disability (including Down syndrome), cardiovascular disorders, kidney and/or liver pathology, other non-specified malignancies and metabolic disorders. Distinction was made between patients with single and multiple underlying disorders. Descriptive statistics were calculated for all available clinical and epidemiological characteristics. Fisher's exact test was used to compare the 2009-2010 pandemic influenza season (week 30 2009 - week 39 2010) and the 2010-2011 influenza season (week 40 2010 - week 39 2011) with respect to binary variables, and Wilcoxon-Mann-Whitney test was used for continuous variables. For both seasons, the pandemic influenza mortality rate per 100, 000 persons was calculated as the number of fatal pandemic influenza cases divided by the total Dutch population (determined on 1 January 2009 and 2010) * 100, 000. For this analysis, the patients were categorised in the following age groups:0-4, 5-14, 15-24, 25-34, ..., 75-84 and ≥ 85 years of age. All statistical analyses were conducted using SAS version 9.2 (SAS Institute).

## Results

A total of 103 fatalities with a laboratory-confirmed influenza A(H1N1) 2009 virus infection were notified: 65 in the 2009-2010 pandemic season and 38 in 2010-2011 influenza season. No pregnancy-related pandemic influenza deaths were notified in either season. In 2009-2010, 61 of the 65 patients (94%) were admitted to hospital, 44 of them (68%) were admitted to the ICU, and 28 (43%) required mechanical ventilation because of respiratory insufficiency. In 2010/2011, 37 of the 38 fatalities (97%) were admitted to hospital, 27 of them (71%) to the ICU, and 23 (61%) required mechanical ventilation. No statistical significant differences between the two seasons were found for these variables.

### Age distribution

**Figure 1:**
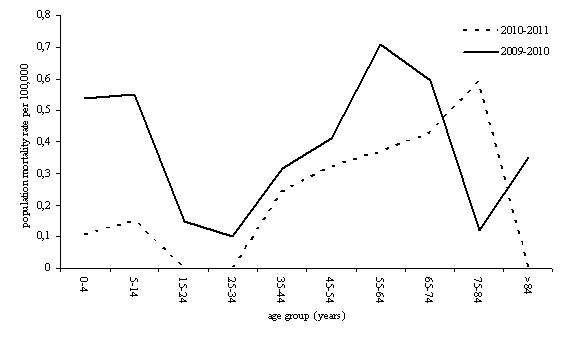
**Population mortality rates for laboratory-confirmed influenza A(H1N1) 2009 virus infection per 100, 000 persons for the pandemic season 2009-2010 and the following season 2010-2011**.

The mean age of the deceased patients in 2009/2010 was lower compared to that in 2010/2011, respectively 41 and 53 years (p = 0.02). Figure 1 shows the mortality rate per age group based on the total Dutch population in 2009 and 2010. During the pandemic season, the population mortality rates peaked in children aged between 0 and 15 years of age and in persons aged between 55 and 64 years. In the 2010-2011 influenza season, the first peak was considerably lower, while the second peak shifted to persons aged between 75 and 84 years.

### Clinical presentation

Data on clinical presentation was available for 42 of the 65 fatalities (65%) during the pandemic seasons and for 28 of the 38 (74%) during the 2010-2011 season (table 1). In both seasons, fatal cases presented mainly with respiratory symptoms (41%), including acute respiratory distress syndrome (ARDS), followed by systemic symptoms (17%).

**Table 1:** Clinical presentation reported for the deceased patients with a laboratory-confirmed influenza A(H1N1)2009 virus infection, the Netherlands 2009-2011 (n = 103)

	Pandemic season 2009-2010 N = 65		Influenza season 2010-2011 N = 38	
Clinical presentation	n	(%)	n	(%)
respiratory, including ARDS*	26	(40)	16	(42)
systemic	11	(17)	7	(18)
neurological	3	(5)	1	(3)
super infection	2	(3)	2	(5)
cardiovascular	-		2	(5)
unknown	23	(35)	10	(26)

* ARDS = acute respiratory distress syndrome

Immunological and respiratory disorders were the most commonly reported underlying medical conditions in the pandemic season, for respectively 23 (35%) and 22 (34%) of the 65 fatalities (table 2). In the 2010-2011 season, cardiovascular disorders and absence of medical underlying conditions were most common, respectively for 12 (32%) and 10 (26%) of the 38 deaths. Underlying immunological disorders were more common in the 2009-2010 compared to the 2010-2011 season (p = 0.02), while cardiovascular disorders were significantly more common in the 2010-2011 season (p = 0.005). Overall, multiple underlying conditions were reported for 27 of the 103 cases (26%). Particularly, intellectual disability (100%), cardiovascular (76%) and metabolic disorders (75%) were found in combination with other underlying conditions (data not shown).

**Table 2:** Underlying medical conditions of deceased patients with a laboratory-confirmed influenza A(H1N1)2009 virus infection, the Netherlands 2009-2011 (n = 103)

	Pandemic season 2009-2010 N = 65		Influenza season 2010-2011 N = 38		
Underlying medical condition*	n	(%)	n	(%)	p
None**	10	(15)	10	(26)	0.3
Respiratory disorder	22	(34)	7	(18)	0.1
Immunological disorder^#^	23	(35)	5	(13)	**0.02**
Neurological disorder	9	(14)	4	(10)	0.9
Intellectual disability^##^	8	(12)	2	(5)	0.4
Cardiovascular disorders	5	(8)	12	(32)	**0.005**
Kidney/liver disorders	4	(6)	3	(8)	0.9
Malignancies^###^	2	(3)	3	(8)	**0.5**
Metabolic disorders	1	(2)	3	(8)	**0.3**
Multiple underlying disorders	17	(26)	10	(26)	> 0.9

* Patients with multiple underlying disorders are counted for every underlying medical condition. For one patient underlying medical condition was unknown.

** In one patient a group A streptococcal infection was confirmed after autopsy.

^# ^Including haematological malignancies.

^## ^Including Down syndrome.

^### ^Other non-specified malignancies.

## Discussion

The peak in mortality rates in persons aged between 55 and 64 years observed during the 2009-2010 pandemic, shifted to older age groups in the 2010-2011 influenza season. Furthermore, the peak in mortality rates in children younger than 15 years of age decreased considerably.

The decline of the peak in children might partly be explained by immunity in the youngest age groups, possibly related to high attack rates of influenza A(H1N1) 2009 virus in children during the pandemic season or to persisting vaccine-induced immunity [14-16]. Although the infection attacks rates during the pandemic season were very low in the older adults (≥ 40 years), the shift of the peak in mortality rates towards older age groups observed in our study might indicate increased circulation of the virus in the 2010-2011 influenza season in these age groups [14].

A shift of the age-specific mortality pattern similar to that observed in our study is also described for the post-pandemic seasons following the three pandemics in the 20^th ^century. During each of these earlier pandemics, persons younger then 65 years of age initially accounted for a high proportion of influenza-related deaths, followed by a declining proportion of deaths in the post-pandemic seasons [17]. Simonsen et al. [17] hypothesised that younger persons may retain long-lasting immunity better than older persons after exposure to a new influenza virus subtype.

Recent studies on risk factors for influenza A(H1N1) 2009 deaths concluded that the majority of severe pandemic cases as well as fatalities had underlying medical conditions as previously also associated with severe seasonal influenza [4,8,18-22]. Our results are in line with previous studies in which respiratory disorders and immunosuppressive conditions were frequently reported as underlying diseases [4-6,18,21]. Furthermore, neurological disorders have been reported to be common underlying diseases in fatal pandemic influenza cases, especially in children and young adults [5,19]. Patients with neurological and neuromuscular disease have also been recognized as high-risk group for severe disease from seasonal influenza [23].

Our study showed a noticeable number of deceased patients (10%) with intellectual disability. Pérez-Padilla et al. [24] recently showed that Down syndrome was associated with adverse outcomes in cases of influenza-like illness (ILI) and severe acute respiratory illness (SARI) during the first months of the outbreak A(H1N1) 2009 influenza virus. All intellectual disabled patients in our study also had other chronic underlying conditions, making it impossible to assess the specific role of intellectual disability as a risk factor for fatal influenza.

Although it has been reported that the A(H1N1) 2009 influenza virus caused severe illness and death in pregnant and postpartum women [25-27], as observed for seasonal influenza, no pregnancy-related pandemic influenza deaths were notified in the Netherlands. As we noted for intellectual disorders, also for pregnancy fatalities it is important to verify whether other chronic underlying conditions are present.

The relatively high number of fatalities with underlying cardiovascular disorders in the 2010-2011 influenza season might be associated with the shift of the mortality rates to elderly persons, since cardiovascular disorders are generally more common in elderly persons. Because of the relatively small numbers of fatalities, it is not possible to compare the differences in underlying conditions between the two seasons adjusted by age.

There remains a possibility that fatal case ascertainment is incomplete because of underreporting and -diagnosing. Especially in patients with severe underlying diseases and elderly, the generally non-specific symptoms may not have been recognized as being caused by influenza A(H1N1) 2009 virus infection. This is reflected by the fact that pandemic influenza was reported as contributing cause of death in some patients instead of the main cause of death. Moreover, this might also partly explain the relatively high mortality in patients with underlying immunological disorders in the 2009-2010 pandemic season compared to the 2010-2011 influenza season. It is plausible that testing for influenza was more common during the pandemic season because of heightened attention, particularly in patients with severe underlying diseases like immunological disorders.

To improve completeness of reporting in the hectic pandemic season, additional information on underlying conditions was actively collected where not available, which might have caused some information bias. Another limitation of this study is the lack of reliable historical records on deaths related to laboratory-confirmed influenza. Although deaths associated with laboratory-confirmed A(H1N1) 2009 virus infection were notifiable during the 2009-2010 and 2010-2011 seasons, clinical influenza diagnoses are generally not laboratory-confirmed during seasonal influenza epidemics. Nevertheless, estimates of the burden of seasonal influenza show that about 90% of influenza-associated deaths occur in persons aged 65 years and older [9]. This is obviously different from the age specific mortality pattern seen during the 2009 and previous pandemics.

## Conclusions

The maintenance of the mandatory notification of deaths associated with laboratory-confirmed influenza A(H1N1) 2009 made it possible to compare the fatal cases during the 2009-2010 pandemic season with that during the 2010-2011 influenza season. The mortality pattern in the 2010-2011 season still resembles the pandemic season with a peak in relatively young age groups, but concurrently shows a clear shift towards the seasonal pattern, as also described for previous pandemics in the 20^th ^century.

## Competing interests

**No financial support **All authors: no financial support

**Potential conflict of interest **All authors: no conflicts

## Authors' contributions

AvG designed the study, analysed and interpreted the data and drafted the manuscript. RR participated in the collection and interpretation of the data and editing the manuscript. IF participated in the interpretation of the data, performing the statistical analyses and editing the manuscript. AM participated in the collection and interpretation of the data, in editing the manuscript and was responsible for the virological assays. LI participated in the collection and interpretation of the data, and editing the manuscript. AT participated in the collection and interpretation of the data, and editing the manuscript. MvdS participated in the design of the study, interpretation of the data, performing the statistical analyses and editing the manuscript.

All authors read and approved the final manuscript.

## Pre-publication history

The pre-publication history for this paper can be accessed here:

http://www.biomedcentral.com/1471-2458/11/758/prepub
